# Effects of a Cool-Down after Supramaximal Interval Exercise on Autonomic Modulation

**DOI:** 10.3390/ijerph19095407

**Published:** 2022-04-29

**Authors:** Jason C. Parks, Erica M. Marshall, Stacie M. Humm, Emily K. Erb, J. Derek Kingsley

**Affiliations:** 1 Kinesiology, State University of New York at Cortland, Cortland, NY 13045, USA; 2 Exercise Science, Florida Southern College, Lakeland, FL 33801, USA; emarshall@flsouthern.edu; 3 Exercise Science and Exercise Physiology, Kent State University, Kent, OH 44242, USA; shumm2@kent.edu (S.M.H.); eerb4@kent.edu (E.K.E.); jkingsle@kent.edu (J.D.K.)

**Keywords:** heart rate variability, heart rate complexity, active recovery, passive recovery, lactate

## Abstract

Supramaximal interval exercise alters measures of autonomic modulation, while a cool-down may speed the recovery of vagal modulation. The purpose of this study was to compare the effects of a cool-down (pedaling a cycle ergometer at 50 rpm against a resistance of 45 W) versus passive recovery (no pedaling) after supramaximal interval exercise on autonomic modulation. Sixteen moderately active individuals (Mean ± SD: 23 ± 3 years (men: *n* = 10; women: *n* = 6) were assessed for autonomic modulation at Rest, and 15 (R15), 30 (R30), 45 (R45) and 60 (R60) min following supramaximal interval exercise. Linear measures of autonomic modulation included natural log (ln) total power (lnTP), high-frequency power (lnHF), the ratio of low frequency (LF) to HF ln(LF/HF) ratio, root mean square of successive differences between normal heartbeats (lnRMSSD), while non-linear measures included sample entropy (SampEn) and Lempel–Ziv entropy (LZEn). Two-way repeated ANOVAs were used to evaluate the main effects of condition (cool-down, passive recovery) across time (Rest, and R15, R30, R45 and R60). There were significant (*p* ≤ 0.05) condition by time interactions for SampEn and LZEn, such that they decreased at 15, 30, 45 and 60 min during passive recovery compared to Rest, with the recovery of SampEn and LZEn by 60 and 45 min, respectively, during cool-down. There were significant (*p* ≤ 0.05) main effects of time for lnTP, lnHF and lnRMSSD, such that lnTP, lnHF and lnRMSSD were attenuated, and lnLF/HF ratio was augmented, at all recovery times compared to Rest. These data demonstrate that a cool-down increases the recovery of nonlinear measures of vagal modulation within 45–60 min after supramaximal interval exercise, compared to passive recovery in moderately active individuals.

## 1. Introduction

Supramaximal interval exercise is a popular mode of exercise used in healthy individuals [1,2]. Supramaximal interval exercise involves the control of intensity and rest intervals during repeated bouts to increase the volume of supramaximal work while reducing total exercise time, resulting in a time-efficient means to induce positive adaptations in skeletal muscle [3]. Researchers have demonstrated that acute bouts of supramaximal interval exercise may result in transient effects on heart rate (HR) and autonomic modulation measured via linear methods of heart rate variability (HRV) or nonlinear methods using heart rate complexity (HRC). The transient effects on HR and autonomic modulation after supramaximal interval exercise were demonstrated by increases in HR and sympathovagal dominance [4,5], with reductions in linear and nonlinear measures of vagal modulation immediately following [6], and for at least 60 min after the supramaximal exercise [4,5]. This information is important, considering the transient risk of a cardiovascular event may increase after vigorous exertion [7], and reductions in measures of vagal modulation have been observed prior to the onset of atrial fibrillation [8,9]. Taken together, it is clear that supramaximal interval exercise increases HR and sympathovagal dominance with concomitant reductions in vagal modulation, at least temporarily [4], but the data are sparse.

The American College of Sports Medicine recommends a cool-down consisting of at least 5–10 min of light-to-moderate aerobic and muscular endurance exercise to facilitate the gradual recovery of HR and blood pressure (BP), and to aid in the clearance of metabolites, such as hydrogen ions and blood lactate after vigorous exercise [10], with no recommendations following supramaximal exercise. Data have demonstrated that a cool-down may increase vagal modulation, thereby attenuating HR after continuous vigorous-intensity exercise [11], but no such data exist regarding supramaximal interval exercise. However, based on the data, the responses to both continuous vigorous-intensity exercise and supramaximal interval exercise, in terms of cardiovascular recovery, appear to be relatively similar [2,4,12,13]. Given that the cardiovascular responses to both intensities appear similar, it is likely that the prescription of a cool-down after supramaximal interval exercise may be beneficial in promoting autonomic recovery. Despite ACSM’s recommendation of a cool-down after vigorous exercise, not one of the existing studies examining the effects of supramaximal interval exercise on the cardiovascular system included a cool-down of at least five min in their study protocol [4,5,13,14], which may have resulted in a misleading or inaccurate interpretation of the data; thus more data on a cool-down following supramaximal exercise is both pertinent and necessary.

Understanding how the use of a cool-down influences HR and autonomic modulation after supramaximal exercise may be an important step in improving the exercise prescription. Therefore, the purpose of this study was to determine whether utilizing a cool-down after supramaximal interval exercise has any effect on the recovery of HR and autonomic modulation up to 60 min in moderately active individuals, compared to passive recovery. We hypothesized that HR and sympathovagal dominance would significantly increase, and vagal modulation, measured using both linear and nonlinear assessments of autonomic modulation, would significantly decrease for up to 60 min after acute supramaximal interval exercise, compared to rest, regardless of the recovery condition. We also hypothesized that the use of a cool-down would result in the earlier recovery of HR, as well as linear and nonlinear assessments of autonomic modulation within 60 min after exercise.

## 2. Materials and Methods

### 2.1. Participants

Eighteen moderately-active individuals participated in this study. However, sixteen young (20–26 years), healthy, moderately active individuals (men = 12, women = 6) completed this study, as two participants were not able to complete the exercise protocol. Participants were recruited through flyers posted on bulletin boards and through social media. Participants were deemed moderately active based on the Lipid Research Clinics Physical Activity Questionnaire, which consists of four questions and uses a four-point scoring method to determine each participant’s physical activity classification [15]. Participants were excluded from the study if they had known vascular or metabolic disease, uncontrolled hypertension (resting brachial BP ≥ 130/80 mmHg), a recent smoking history (<6 months since last tobacco use), obesity (defined as a body mass index ≥ 30 kg/m^2^), cancer, orthopedic problems, history of blood clots, and/or open wounds. Participants were excluded if they were taking supplements or medications known to affect HR, BP, or vascular function. Inclusionary criteria were self-reported All women were tested during the early follicular phase (days 1–7) of their menstrual cycle. This project was approved by the Institutional Review Board, and data were collected in agreement with the Declaration of Helsinki. All participants were informed of the risks and benefits of the study, and signed an institutionally approved informed consent form prior to the collection of any data.

### 2.2. Study Design

**Figure 1:**
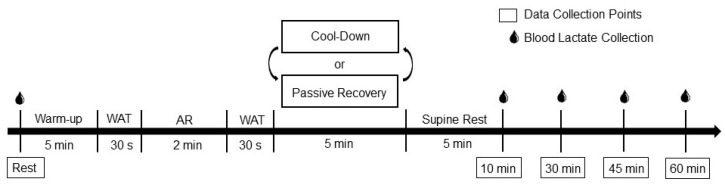
Experimental trial timeline. WAT = Wingate anaerobic test. AR = active recovery.

The present study utilized a repeated measures design in which each participant acted as their own control. Participants reported to the laboratory on three different days. The first visit consisted of an orientation and anthropometric assessment, as well as familiarization with the Wingate anaerobic test (WAT), while the second and third visits were used for data collection. On the data collection days, participants arrived at the laboratory having abstained from strenuous exercise, alcohol, and caffeine for a minimum of 24 h, and food for 3 h prior to testing. The order of the second and third visits were counterbalanced and consisted of two 30 s WATs, separated by a two-minute active recovery (AR) followed by either the cool-down or passive recovery condition (see Figure 1). The cool-down consisted of pedaling a cycle ergometer at 50 rpm against a light resistance of 45 W for 5 min and the passive recovery required the participant to sit upright in a chair for the same period of time. There were at least 72 h between exercise sessions, and participants were asked to refrain from strenuous exercise between sessions. The two exercise sessions were performed during the same time of day (±1 h) for each participant. All testing occurred between the hours of 7 am and 12 pm to control for diurnal variation. Prior to data collection, participants rested in a supine position for 10 min while all instrumentation was applied. Once the resting data were collected, participants performed two WATs. After the second WAT, the participant completed the 5 min cool-down or passive recovery condition, followed by 5 min of supine rest prior to recovery data collection. During recovery, data were collected at Rest and 15, 30, 45, and 60 min after exercise (Figure 1).

### 2.3. Anthropometrics

Height and weight were measured using a stadiometer and balance platform scale, respectively. Height was measured to the nearest 0.1 cm, and weight was measured to the nearest 0.1 lb and then converted to kg.

### 2.4. Autonomic Modulation

Continuous 3-lead electrocardiogram (ECG) signals were collected via a modified CM5 configuration (PowerLab; AD Instruments, Colorado Springs, CO, USA) at a rate of 1000 Hz. HR was determined via the ECG recordings. Respiration rate was set at 12 breaths per min during all data collection. The ECG signals were imported into WinCPRS analyzing software Version 1.162 (Absolute Aliens; Turku, Finland) after visual inspection for noise, ectopic beats, as well as artifacts, and no interpolation was performed. Fast Fourier transform was used to generate spectral power. Linear measures of autonomic modulation (HRV) were assessed in both the frequency and time domains. In the frequency domain, total power (TP) represented the total autonomic activity (6). Low-frequency power (LF) consisted of both sympathetic activity and vagal modulation, with a range between 0.04–0.15 Hz, and high-frequency power (HF) was a measure of vagal modulation, with a range between 0.15 and 0.4 Hz (6). The LF/HF ratio was used to represent sympathovagal dominance [16]. In the time domain, the root mean square of successive differences between normal heartbeats (RMSSD) was used as a measure of vagal modulation [17].

The non-linear measures of autonomic modulation (HRC) were sample entropy (SampEn) and Lempel–Ziv entropy (LZEn). These measures were used to examine the complexity of the R-R intervals over a 5 min epoch, after the removal of the linear trend. SampEn is defined as the probability of similar sequences or successive matches over a short period of time, with a range from 0 to 2 [18]. Values nearer to 0 are indicative of a more predictive signal and values approaching 2 are indicative of a more chaotic signal [18]. LZEn involves counting the number of distinct and repeating patterns within a time sequence [19]. Each distinct pattern, or subsequence, is assigned a symbol and then converted to 0 s and 1 s to form a binary substring. When reading the time sequence from left to right, each time a new substring of consecutive digits is encountered, a new symbol is added [20]. The size of the symbol vocabulary and rate at which distinct symbols occur along a sequence determines the complexity of that sequence [20]. LZEn values further from zero indicate an increase in the complexity of the sequence [21].

### 2.5. Blood Lactate

Blood lactate levels were measured by making a small puncture in the ear lobe using a Unistik 3, single-use safety lancet (Owen Mumford Ltd.; Oxfordshire, UK). A 0.2 µL blood sample was collected and blood lactate concentration was measured with a lactate Plus analyzer (Nova Biomedical; Waltham, MA, USA). The lactate Plus analyzer has been found to be a reliable and accurate alternative to laboratory-based bench top analyzers [22]. Lactate Plus, single use, disposable test strips (Nova Biomedical; Waltham, MA, USA) were used to collect the sample during the study protocol.

### 2.6. Supramaximal Interval Exercise

Prior to completing the supramaximal interval exercise protocol, each participant completed a 5 min warmup on the cycle ergometer pedaling at 50 rpm against a light resistance of 45 W. The supramaximal interval exercise consisted of two, maximal effort, 30 s WATs performed using a mechanically braked cycle ergometer (Monarch Model 894 E; Stockholm, Sweden). The WAT involved pedaling against a constant force [23] and the resistance was set to 7.5% of the participant’s body weight based on previous studies [5,14]. Participants performed 2 min of active recovery (pedaling at 50 rpm against a light resistance of 45 W) between the exercise bouts. After the second and final WAT, participants completed the 5 min cool-down (pedaling at 50 rpm against a light resistance of 45 W) or passive recovery condition (sitting relatively still in a chair). Once these conditions were completed, all participants moved into a supine position for five 5 min prior to the collection of recovery data (see Figure 1). To ensure the safety of the participants, HR and continuous BP were monitored during the exercise bout and the 5 min cool-down or passive recovery conditions (NIBP Nano monitoring system, AD Instruments; Colorado Springs, CO, USA) and sampled at 1000 and 200 Hertz (Hz), respectively.

### 2.7. Statistical Analysis

A 2 × 5 repeated measures analysis of variance (ANOVA) was used to determine the effects of condition (cool-down or passive recovery) across the repeated measure of time (Rest, 15, 30, 45, and 60 min) on HR, autonomic modulation (TP, LF, HF, LF/HF ratio, RMSSD, SampEn and LZEn) and blood lactate after supramaximal interval exercise. Because TP, LF, HF, LF/HF ratio, and RMSSD were not normally distributed, as revealed by the Shapiro–Wilk test, these values underwent log transformation (ln). Paired t-tests were performed for all post hoc comparisons with a Benjamini–Hochberg correction [24] to control for alpha inflation. Partial eta squared (*η_p_*^2^) was used to assess the effect size of each dependent variable [25]. Percent change was calculated using the formula New number—Original number/Original number × 100, but was not run statistically. Significance was set a priori at *p* ≤ 0.05. Values are presented as the mean ± standard deviation (SD). All statistical analyses were completed using IBM SPSS (Version 25, IBM, Armonk, NY, USA). The sample size of 16 participants was based on data collected by Kingsley et al. [14]. The G*Power sample size calculator, version 3.1.9.2 (Heinrich-Heine-Universität Düsseldorf, Dusseldorf, Germany) [26], was used to estimate a minimum sample size of eight to achieve a power of 80% based on an effect size (Cohen’s f) of 1.13 and an alpha of 0.05.

## 3. Results

Participant characteristics are presented in Table 1. Measures of HR and autonomic modulation are presented in Table 2. There were no significant (*p* > 0.05) differences in average power (W/kg) during the WATs, when comparing the cool-down and passive recovery conditions.

There were no significant interactions for HR, lnTP, lnLF, lnHF or lnRMSSD. However, there was a significant condition by time interaction for the lnLF/HF ratio (F_4,79_ = 3.07, *p* = 0.023, *η_p_*^2^ = 0.17), such that there was a significant increase from 15 to 30 min after supramaximal interval exercise during the passive recovery condition, with no significant increase during the cool-down condition. There were significant main effects of time for HR (F_4,79_ = 108.66, *p* ≤ 0.001, *η_p_*^2^ = 0.88) and the ln(LF/HF) ratio (F_4,79_ = 17.68, *p* ≤ 0.001, *η_p_*^2^ = 0.54), such that they were augmented at 15, 30, 45 and 60 min after supramaximal interval exercise compared to Rest, during both conditions. There were significant main effects of time for lnTP (F_4,79_ = 34.94, *p* ≤ 0.001, *η_p_*^2^ = 0.79), lnLF (F_4,__79_ = 59.78, *p* ≤ 0.001, *η_p_*^2^ = 0.80), lnHF (F_4,79_ = 70.48, *p* ≤ 0.001, *η_p_*^2^ = 0.83) (Table 2), and lnRMSSD (F_4,79_ = 75.72, *p* ≤ 0.001, *η_p_*^2^ = 0.84), such that they were attenuated at 15, 30, 45 and 60 min after supramaximal interval exercise compared to Rest, during both conditions.

**Figure 2:**
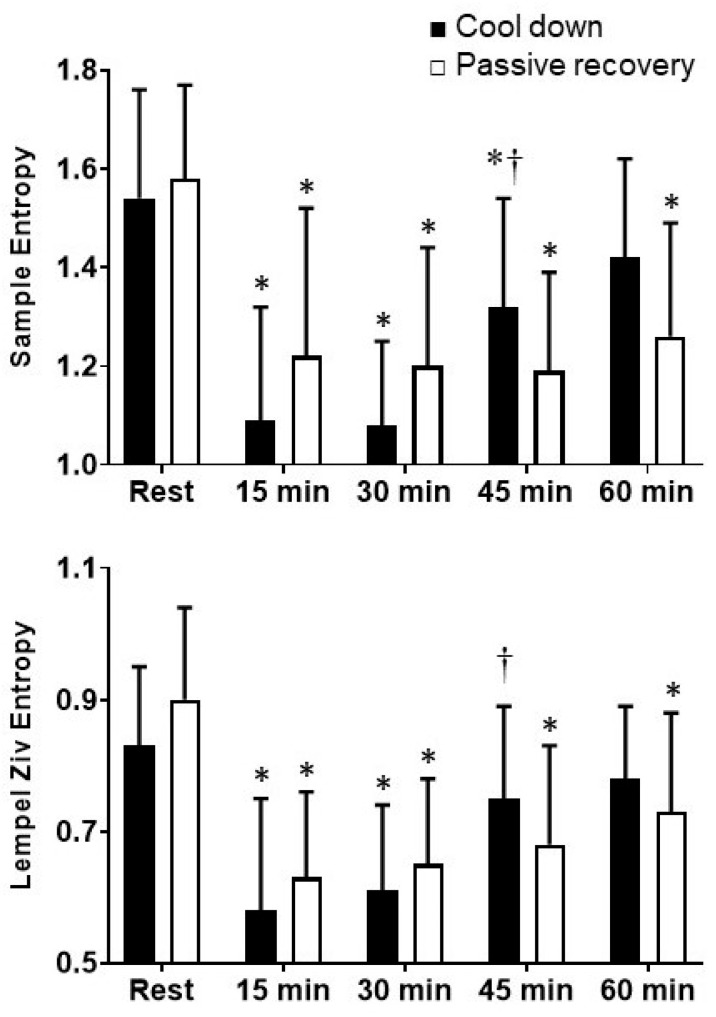
Sample entropy and Lempel–Ziv entropy at Rest and 15, 30, 45 and 60 min during recovery in moderately active individuals. Values are mean ± SD. * Significantly different from Rest (*p* ≤ 0.05), † Significantly different from 30 min (*p* ≤ 0.05).

There were significant condition by time interactions for SampEn (F_4,79_ = 5.79, *p* ≤ 0.001, *η_p_*^2^ = 0.28) and LZEn (F_4,79_ = 2.66, *p* = 0.041, *η_p_*^2^ = 0.15), such that there were significant increases from 30 to 45 min after exercise during the cool-down condition, with no significant increase during the passive recovery condition (Figure 2). There were also significant main effects of time for SampEn (F_4,79_ = 17.43, *p* ≤ 0.001, *η_p_*^2^ = 0.54) and LZEn (F_4,79_ = 18.01, *p* ≤ 0.001, *η_p_*^2^ = 0.55), such that they were attenuated at 15, 30, 45 and 60 min after supramaximal interval exercise during the passive recovery condition, compared to Rest, with the recovery of SampEn and LZEn by 60 and 45 min, respectively, during the cool-down condition (Figure 2).

There was a significant condition by time interaction for blood lactate (F_4,79_ = 15.99, *p* ≤ 0.001, *η_p_*^2^ = 0.50), such that it was significantly lower with a cool-down at 15, 30, 45 and 60 min after supramaximal interval exercise, compared to the passive recovery condition. There was a significant main effect of time for blood lactate (F_4,79_ = 504.81, *p* ≤ 0.001, *η_p_*^2^ = 0.97), such that it was augmented at 15, 30, 45 and 60 min after supramaximal interval exercise compared to Rest during both conditions (Table 2).

## 4. Discussion

The present study sought to compare changes in HR and autonomic modulation using a cool-down versus passive recovery for up to 60 min after an acute bout of supramaximal interval exercise in moderately active individuals. The primary findings were that an acute bout of supramaximal interval exercise increases HR, decreases linear measures of vagal modulation, and increases sympathovagal dominance for up to 60 min with both a cool-down and passive recovery. However, non-linear measures of vagal modulation recover within 45–60 min after supramaximal interval exercise with the use of a cool-down, but remains depressed for at least 60 min with passive recovery. Collectively, these findings indicate that an acute bout of supramaximal interval exercise results in a pronounced loss of vagal modulation, with increases in sympathovagal dominance in moderately active individuals, and that the use of a cool-down may facilitate the earlier recovery of non-linear measures of vagal modulation.

The present study demonstrated that an acute bout of supramaximal interval exercise resulted in significant increases in HR of 55%, 39%, 27% and 22%, compared to Rest, at 15, 30, 45, and 60 min after exercise, respectively, with a cool-down and passive recovery. These results are in contrast with our hypothesis which stated the HR response would be attenuated within 60 min after exercise using a cool-down, compared to passive recovery. The increases in HR are consistent with Millar et al. (2009) who reported significant increases in HR of 87% and 43%, compared to Rest, at 5–20 and 45–60 min, respectively, during recovery from four repeated WATs with no cool-down after the intervals [4]. The results of the present study are also in agreement with Kingsley et al. (2016) who reported increases in HR of 46% at 10–15 min after three repeated WATs with no cool-down after the intervals [14]. Specifically, Kingsley et al. (2016) had participants return to the supine position within one minute of completing their third and final WAT, compared to five min in the present study [14]. The significant increases in HR during recovery from an acute bout of supramaximal interval exercise demonstrated in these studies indicate a loss of vagal modulation. Data from the present study demonstrate that the use of a cool-down after supramaximal interval exercise does not attenuate HR during recovery compared to passive recovery. Collectively, these data demonstrate that an acute bout of supramaximal interval exercise results in the augmentation of HR for up to 60 min after exercise with or without a cool-down.

Our study is the first to examine the effects of a cool-down and supramaximal interval exercise on autonomic modulation in moderately active individuals. Our data demonstrate that supramaximal interval exercise decreases linear measures of vagal modulation, demonstrated by the attenuation of lnHF and lnRMSSD for up to 60 min after acute supramaximal interval exercise, compared to Rest, with a cool-down and passive recovery. These results are in partial agreement with our hypothesis which stated linear measures of vagal modulation would be attenuated for up to 60 min, but would be augmented within 60 min after exercise using a cool-down, compared to passive recovery. There were no significant differences between the conditions. However, the decrease in lnHF of 51%, 41%, 29% and 21% at 15, 30, 45 and 60 min, respectively, compared to Rest, after supramaximal interval exercise in the present study, are in agreement with Millar et al. (2009) who reported decreases in lnHF of 95% and 51%, compared to Rest, at 5–20 and 45–60 min, respectively, after four repeated WATs [4]. The difference in the magnitude of change in lnHF observed between these studies support the findings of Millar et al. (2009) who reported a dose–response relationship between the total volume of supramaximal work and vagal modulation [4]. The study by Millar et al. (2009) also included data from 5–10 min after exercise, which may include a time period of greater vagal withdrawal, compared to 15 min after exercise in the present study. Data from the present study revealed no differences in the lnRMSSD between conditions, but demonstrated a significant decrease of 49%, 38%, 27% and 22%, compared to Rest, at 15, 30, 45 and 60 min, respectively, compared to Rest. To our knowledge, the present study is the first to report lnRMSSD data after acute supramaximal interval exercise. Perkins et al. (2015) reported a significant decrease in RMSSD immediately after supramaximal interval exercise [6]. However, these data were not reported as log transformed and cannot be directly compared with data from the present study. The significant decreases in linear measures of vagal modulation reported in the present study reflect an increase in vagal withdrawal. Taken together, the data from the present study demonstrate that acute supramaximal interval exercise, with and without a cool-down, decreases linear measures of vagal modulation for up to 60 min.

Our data demonstrated significant increases in the ln(LF/HF) ratio at 15, 30, 45 and 60 min, respectively, after an acute bout of supramaximal interval exercise, compared to Rest, with or without a cool-down. The findings of the present study support the work of Millar et al. (2009) who also reported significant increases in the ln(LF/HF) ratio compared to Rest for up to 60 min after four repeated WATs [4]. Decreases in lnLF and lnHF after exercise were reported in the present study and by Millar et al. (2009), supporting the concept that the LF/HF ratio is a measure of sympathovagal dominance as opposed to balance [16]. In both studies, the magnitude of vagal withdrawal was greater than the decrease in lnLF, mediating the increase in the LF/HF ratio. Collectively, these data suggest that an acute bout of supramaximal interval exercise augments sympathovagal dominance for up to 60 min after exercise, regardless of whether a cool-down is utilized.

Data from the present study demonstrate that supramaximal interval exercise, with passive recovery, decreases nonlinear measures of vagal modulation, as demonstrated by the attenuation of SampEn and LZEn for up to 60 min after acute supramaximal interval exercise compared to Rest. However, the use of a cool-down after exercise resulted in the recovery of SampEn and LZEn within 60 and 45 min, respectively, which supported our hypothesis. SampEn and LZEn are measures of the complexity of vagal modulation. Data from the present study demonstrate decreases in SampEn with a passive recovery of 23%, 24%, 25% and 20% at 15, 30, 45 and 60 min after exercise, respectively. The attenuation of SampEn when utilizing passive recovery in the present study supports the findings of Millar et al. (2009), who demonstrated a significant decrease in SampEn of 52% and 40% at 5–20 and 45–60 min, respectively, compared to Rest, after four repeated WATs, in recreationally active men [4]. Data from the present study demonstrated that when utilizing a cool-down, there were significant decreases in SampEn of 29%, 30% and 14% at 15, 30 and 45 min, respectively, with recovery at 60 min after exercise. To our knowledge, the present study is the first to report changes in LZEn after supramaximal interval exercise. Data demonstrated significant decreases in LZEn of 30%, 28%, 24% and 19% at 15, 30, 45 and 60 min, respectively, when utilizing passive recovery. However, when utilizing a cool-down, LZEn decreased by 30% at 15 and 30 min, but recovered within 45 min after exercise. The early recovery of vagal complexity with the use of a cool-down in the present study may reflect the decreased metabolic demand, as demonstrated by significantly lower blood lactate levels during and after exercise compared to passive recovery. Taken together, these data suggest that supramaximal interval exercise attenuates the complexity of vagal modulation for up to 60 min with passive recovery, but utilizing a cool-down results in the recovery of vagal modulation within 45–60 min. This is important because transient decreases in vagal modulation after vigorous exertion may result in increased cardiovascular risk [7,8].

As expected, there were significant increases in blood lactate levels for up to 60 min after exercise, compared to Rest under both conditions, with significantly lower levels when a cool-down was utilized. The increases in blood lactate in the present study are consistent with the findings of Stuckey et al. (2012) who demonstrated a significant increase in blood lactate concentration in recreationally active men for up to 60 min after four repeated WATs [5]. The performance of a cool-down was demonstrated to accelerate lactate clearance after exercise [27]. It is well known that the enhanced buffering capacity during light-to-moderate exercise resulting from cardiovascular activation improves lactate clearance.

There were some limitations to the present study. The results of this study may be limited to moderately active individuals. The prescription of supramaximal interval exercise may limit the ability to generalize the results in comparison to modalities performed at lower intensities. Supramaximal interval exercise may also be performed at an intensity that is too high for some individuals with cardiovascular or metabolic disease. In addition, although inclusionary criteria for participation in this study were met using validated questionnaires, responses were self-reported.

## 5. Conclusions

These data demonstrate that the use of a cool-down after an acute bout of supramaximal interval exercise results in the early recovery of nonlinear measures of vagal modulation, indicating an increase in vagal modulation within 45–60 min after exercise when compared to passive recovery. These data also demonstrate that an acute bout of supramaximal interval exercise results in significant increases in HR and sympathovagal dominance, with concomitant decreases in linear measures of vagal modulation for at least 60 min after exercise compared to Rest, with or without a cool-down. Future research should increase the duration of the cool-down protocol after vigorous exercise to determine whether a longer cool-down period facilitates the recovery of linear measures of vagal modulation and accelerates the recovery of nonlinear measures of vagal modulation.

## Figures and Tables

**Table 1:** Participant characteristics (*n* = 16).

	Indicators
Age (y)	23 ± 3
Height (m)	1.76 ± 0.10
Weight (kg)	74.6 ± 13.7
BMI (kg·m^2^)	24.0 ± 2.7

Data presented are mean ± SD. BMI = body mass index.

**Table 2:** Heart rate, autonomic variables, and blood lactate at Rest and during recovery from supramaximal interval exercise in moderately active individuals (*n* = 16).

	Cool Down					Passive Recovery				
	Rest	15 min	30 min	45 min	60 min	Rest	15 min	30 min	45 min	60 min
**Heart rate (bpm)**	58 ± 8	91 ± 9 *	81 ± 9 *	73 ± 9 *	71 ± 8 *	59 ± 8	90 ± 10 *	82 ± 10 *	75 ± 9 *	72 ± 10 *
**Total power (ln ms^2^)**	8.5 ± 0.9	5.8 ± 0.9 *	6.9 ± 0.9 *	7.5 ± 1.0 *	7.6 ± 1.0 *	8.8 ± 0.9	5.9 ± 1.1 *	6.9 ± 1.2 *	7.7 ± 1.1 *	7.8 ± 0.9 *
**LF (ln ms^2^)**	6.8 ± 0.6	4.2 ± 0.7 *	5.4 ± 0.9 *	6.1 ± 1.2 *	6.2 ± 0.8 *	7.3 ± 0.9	4.1 ± 0.9 *	5.6 ± 1.2 *	6.1 ± 0.7 *	6.2 ± 0.6 *
**HF (ln ms^2^)**	8.0 ± 1.0	3.8 ± 1.3 *	4.8 ± 1.8 *	5.8 ± 1.6 *	6.4 ± 1.4 *	8.0 ± 1.1	4.1 ± 1.4 *	4.7 ± 1.6 *	5.7 ± 1.6 *	6.1 ± 1.5 *
**LF/HF ratio (ln)**	3.5 ± 0.6	5.1 ± 0.9 *	5.2 ± 1.2 *^‡^	4.9 ± 1.1 *	4.4 ± 1.0 *	3.9 ± 0.7	4.7 ± 1.0 *	5.5 ± 1.1 *	5.1 ± 1.1 *	4.7 ± 1.1 *
**RMSSD (ln ms)**	4.5 ± 0.5	2.3 ± 0.7 *	2.9 ± 0.8 *	3.4 ± 0.8 *	3.6 ± 0.7 *	4.5 ± 0.6	2.3 ± 0.9 *	2.8 ± 0.8 *	3.3 ± 0.8 *	3.6 ± 0.8 *
**Blood lactate (mmol)**	0.6 ± 0.2	11.1 ± 1.8 *^#^	6.7 ± 1.7 *^#^	4.2 ± 1.1 *^#^	2.8 ± 0.8 *^#^	0.7 ± 0.3	13.0 ± 2.0 *	8.3 ± 2.2 *	5.2 ± 1.4 *	3.3 ± 1.0 *

Data presented are mean ± SD. * Significantly different from Rest (*p* ≤ 0.05), ^‡^ Significantly different from 15 min (*p* ≤ 0.05), ^#^ Significantly different from passive recovery (*p* ≤ 0.05). HF = high-frequency power; LF = low-frequency power; RMSSD = root mean square of successive differences between normal heart beats.

## Data Availability

Data presented in this study are available upon request from the corresponding author. The data are not publicly available due to privacy/ethical concerns.
